# Neointimal hyperplasia persists at six months after sirolimus-eluting stent implantation in diabetic porcine

**DOI:** 10.1186/1475-2840-6-16

**Published:** 2007-06-05

**Authors:** Qi Zhang, Lin Lu, LiJin Pu, RuiYan Zhang, Jie Shen, ZhengBing Zhu, Jian Hu, ZhenKun Yang, QiuJin Chen, WeiFeng Shen

**Affiliations:** 1 Department of Cardiology, Ruijin Hospital, Shanghai Jiaotong University School of Medicine, Shanghai 200025, PR China

## Abstract

**Background:**

Observational clinical studies have shown that patients with diabetes have less favorable results after percutaneous coronary intervention compared with the non-diabetic counterparts, but its mechanism remains unclear. The aim of this study was to examine the changes of neointimal hyperplasia after sirolimus-eluting stent (SES) implantation in a diabetic porcine model, and to evaluate the impact of aortic inflammation on this proliferative process.

**Methods:**

Diabetic porcine model was created with an intravenous administration of a single dose of streptozotocin in 15 Chinese Guizhou minipigs (diabetic group); each of them received 2 SES (Firebird, Microport Co, China) implanted into 2 separated major epicardial coronary arteries. Fifteen non-diabetic minipigs with SES implantation served as controls (control group). At 6 months, the degree of neointimal hyperplasia was determined by repeat coronary angiography, intravascular ultrasound (IVUS) and histological examination. Tumor necrosis factor (TNF)-α protein level in the aortic intima was evaluated by Western blotting, and TNF-α, interleukin (IL)-1β and IL-6 mRNA levels were assayed by reverse transcription and polymerase chain reaction.

**Results:**

The distribution of stented vessels, diameter of reference vessels, and post-procedural minimal lumen diameter were comparable between the two groups. At 6-month follow-up, the degree of in-stent restenosis (40.4 ± 24.0% vs. 20.2 ± 17.7%, p < 0.05), late lumen loss (0.33 ± 0.19 mm vs. 0.10 ± 0.09 mm, p < 0.001) by quantitative angiography, percentage of intimal hyperplasia in the stented area (26.7 ± 19.2% vs. 7.3 ± 6.1%, p < 0.001) by IVUS, and neointimal area (1.59 ± 0.76 mm^2 ^vs. 0.41 ± 0.18 mm^2^, p < 0.05) by histological examination were significantly exacerbated in the diabetic group than those in the controls. Significant increases in TNF-α protein and TNF-α, IL-1β and IL-6 mRNA levels were observed in aortic intima in the diabetic group.

**Conclusion:**

Neointimal hyperplasia persisted at least up to 6 months after SES implantation in diabetic porcine, which may be partly related to an exaggerated inflammatory response within the blood vessel wall. Our results provide theoretical support for potential direct beneficial effects of anti-diabetic and anti-inflammation medications in reducing the risk of restenosis after stenting.

## Background

Diabetes mellitus has now been a worldwide epidemic and is growing as a public problem. Coronary artery disease is highly prevalent and becomes the major cause of morbidity and mortality in diabetic patients [1-4]. Meanwhile, approximately 25% of patients undergoing percutaneous coronary intervention have diabetes [5,6], and diabetes has been repeatedly identified as an independent predictor of restenosis after coronary balloon angioplasty or bare-metal stent implantation [7]. The advent and clinical application of drug-eluting stents have been regarded as the third milestone in interventional cardiology. Numerous animal experiments and clinical trials have demonstrated the safety and efficacy of sirolimu-eluting stents (SES) in treating patients with coronary artery disease, by dramatically inhibiting neointimal hyperplasia and reducing incidence of in-stent restenosis and consequently target lesion revascularization, as comparing to the bare-metal stents [8,9]. However, the beneficial effects of SES remain less favorable for diabetic patients than for their non-diabetic counterparts [10,11], and repeat revascularization in diabetic patients continues to be substantially higher [5,12]. The high risk features of diabetic patients with coronary artery disease may include hyperglycemia, insulin resistance, dyslipidemia, inflammation, and thrombophilia [13,14]. The interaction between diabetes and coronary disease is intricate and still needs to be elucidated and focused by both clinicians and basic researchers. Animal models were widely used in diabetes research, but most experiments were done on rodents for various purposes [15]. Large animal models may be more reliable to reflect the clinical status of diabetes, due to their resemblance to human diabetes and diabetic complications in physiology and pathophysiology [16]. In this study, we tested the concept that vascular response was exaggerated in diabetic porcine even after implantation of drug-eluting stents, and activation of cytokines in the blood vessel wall was involved in sustaining proinflammatory mechanisms leading to neointimal proliferation.

## Methods

### Establishment of diabetic model

Fifteen juvenile Chinese Guizhou minipigs (male, aged mean 5 months) obtained from Shanghai Jiaotong University Agriculture College, were raised in separated pens under controlled conditions in the Department of Animal for Scientific Research, Jiaotong University School of Medicine. All minipigs had normal day-night cycle, and room temperature was kept between 18 to 25°C with continuous air changing. Diabetic model was made using intravenous injection of streptozotocin (STZ) by the method described previously [16,17]. Briefly, STZ (S0130, Sigma) was administrated intravenously through an indwelling catheter at a dose of 125 mg/kg after dissolving in sodium citrate buffer (pH 4.7). Elevated blood glucose levels were always observed at the third or fourth day after STZ administration using one-touch SureStepPlus instrument (Johnson & Johnson Inc. USA). Insulin therapy was initiated, if necessary, to maintain a fasting glucose level below 10 mmol/l [18]. Novolin^@ ^30R (Novo Nordisk, Denmark) was given subcutaneously in 14 (93.3%) animals with an average dose of 6.8 ± 2.6 U in the morning and 4.5 ± 1.6 U in the afternoon before meals.

Another 15 age-matched male minipigs without diabetes served as controls (control group). The fasting blood glucose level and body weight at baseline were similar. At 6 months, diabetic animals had significantly lower body weight and increased fasting blood glucose level even after exogenous insulin treatment [18].

### Intracoronary stenting and IVUS imaging

Coronary angiography and stent implantation were performed after stabilizing blood glucose level in diabetic group (mean 12 ± 4 days after STZ administration). Aspirin (Bayer, 300 mg/d) and ticlopidine (Sanofi-Aventis, 250 mg/d) were given at least 2 days before catheter procedures. All animals were sedated with intramuscular ketamine hydrochloride (20 mg/kg) and midazolam (1 mg/kg). After tracheal intubation, anesthesia was induced with mechanical ventilation of oxygen and isoflurane. Right femoral artery was exposed, a 6F arterial sheath was inserted and 200 IU/kg heparin was given via the sheath immediately. A 6F Amplatz right coronary guiding catheter (Cordis, USA) was usually used to engage both right and left coronary arteries. For each animal, 2 Firebird SES (Microport Co, Shanghai, China) with 18 mm in length and 2.5 mm or 3.0 mm in diameter according to the size of reference vessels were implanted with the stent-to-vessel ratio of 1.1~ 1.2:1 [19]. The stent was polymer-based and coated with sirolimus (9 μg/mm), and its efficacy and safety in inhibiting neointimal hyperplasia have been demonstrated by animal experiments and clinical studies [8,20]. After procedure, the accessed femoral artery was ligated, and the animal was extratubated once spontaneous respiratory recovery occurred. Continuous electrocardiographic and blood pressure monitoring was made during the procedure. Aspirin and ticlopidine were continued to the end of study in all animals.

Repeat angiography was performed at 6 months through the counterlateral femoral artery. Meticulous care was taken to obtain coronary angiography at similar projections, as possible. Intravascular ultrasound (IVUS) imaging was made to evaluate the degree of neointimal hyperplasia in all stented vessels. All IVUS images were acquired with automated pullback at 0.5 mm/s after intracoronary nitrates using a commercially available imaging system (Galaxy II, Boston Scientific).

### Image analysis

Offline quantitative analysis of procedural, post-procedural, and 6-month follow-up angiography was performed by an independent core laboratory (TERRA, GE, USA). All imaging analyses included the stented segment as well as their margins, 5 mm proximal and distal to the stent. Late lumen loss was defined as the difference between the minimal lumen diameter immediately after procedure and at 6 months. Restenosis was defined as a reduction of 50% or more of the lumen diameter.

Volumetric IVUS analysis was made with the commercially available software (VIVI Viewer 1.6, Beijing JX Digital Wave Co.) according to protocols previously validated [21]. In brief, vessel, stent, lumen, and neointimal volumes were computed for the stented segment as well as its margins 5 mm distal and proximal to the stent. Percentage of intimal hyperplasia was defined as neointimal volume divided by stent volume.

### Histological assessment

After completion of follow-up angiography and IVUS imaging, an additional dose of 10000 IU heparin was given, and all animals were then euthanized and the hearts were excised. Meticulous care was taken to isolate stented coronary segment (including at least 10 mm of both ends), and the stented coronary vessel was processed according to the standard method described previously [22]. The final vessel sections were stained with hematoxylin eosin. Lumen area, area within the internal elastic lamina (IEL) and maximal intimal thickness were computed (Leica-Qwin software) under the microscope (Olympus-AH2). Neointimal area was determined by IEL minus lumen area. Corrected percentage of stenosis was obtained by dividing percent stenosis by injury score (IS), which was evaluated as: 0 = strut in intima but not in contact with IEL; 1 = strut in contact with but not penetrating IEL; 2 = strut penetrating IEL, in contact with media but not contacting external elastic lamina (EEL); 3 = strut contacting but not penetrating EEL; and 4 = strut penetrating EEL and in contact with adventitia [23].

### Determination of inflammatory factors

Secretary proteins in aortic intima conditioned medium were examined for TNF-α expression by Western blotting in order to investigate inflammatory status, because anti-human TNF-α antibody worked on porcine TNF-α. The levels of TNF-α, IL-1β and IL-6 mRNA in aortic intima were evaluated using reverse transcription and polymerase chain reaction (RT-PCR). Briefly, total RNA of aortic intima was extracted using kits (Qiagen, USA) and then quantified. RT-PCR for TNF-α, IL-1β and IL-6 mRNA amplification was performed with primes (Table 1). Cytokine mRNA levels were analyzed by normalizing with GAPDH mRNA expression. Incubation of tissue explants (aortic intima) in RPMI medium with 1% PSA and 2% HEPES supplement was made under sterile conditions (37°C, 5% CO_2_) for 24 hours. The proteins in conditioned medium were quantified and subjected to SDS-PAGE, and transferred to a polyvinylidene difluoride membrane (Millipore, USA). Equal loading was verified with Ponceau Red staining on the membranes following protein transfer. The membrane was incubated with antibody against porcine TNF-α (sc-8301, Santa Cruz, USA) and immunodetection was performed using ECL Western blotting detection reagents (Amersham Biosciences, USA).

**Table 1:** Primers for TNF-α, IL-1β and IL-6 mRNA amplification

Target gene	Forward primer (5'-3')	Reverse primer(5'-3')
TNF-α	ggctgtacctcatctactcc	cagcaaagtccagatagtcg
IL-1β	gatgaggagtatgagagcga	gacaggcttatgttctgcttg
IL-6	gtgagaagtatgagaagtgtga	gcaggatgagaatgatctttg
GAPDH	acgaccatggagaaggctg	tcgtacgaggaaatgagct

### Statistical analysis

Data are expressed as mean ± standard deviation (SD). Comparisons between groups were made by student *t *test or ANOVA when appropriate. SPSS for Windows 13.0 (SPSS Inc., Chicago, Illinois) software was used for statistical analysis. A value of p < 0.05 was considered significant for evaluating difference between the two groups.

## Results

### Procedural characteristics

Two animals in diabetic group and one in control group developed ventricular fibrillation during stent implantation, which was successfully resuscitated by direct electrical shock. All stents were successfully deployed into the coronary vessels. The distribution of stented vessels and reference vessel diameter prior to the procedure and minimal lumen diameter immediately post stenting were similar in the two groups (Table 2, all P > 0.05). During follow-up, 2 animals experienced pulmonary infection which was treated successfully with intramuscular antibiotics (Penicillin) administration, and all survived to the end of the study.

**Table 2:** Angiographic and procedural features

	Diabetic group (n = 15)	Control group (n = 15)
LAD	14	13
LCX	3	4
RCA	13	13
Reference vessel diameter (mm)	2.59 ± 0.32	2.71 ± 0.27
Stent deploy pressure(atm)	13.4 ± 3.0	14.5 ± 3.7
Final MLD(mm)	3.04 ± 0.30	3.18 ± 0.22
Stent to vessel ratio	1.11 ± 0.12	1.18 ± 0.05

LAD = left anterior descending artery, LCX = left circumflex artery, RCA = right coronary artery, MLD = minimal lumen diameter

### Angiographic and IVUS features

At 6-month follow-up, overall in-stent restenosis rate was higher in the diabetic group, but did not reach statistical significance level compared to control group (23.3% vs. 6.7%, p = 0.15). Angiographic minimal lumen diameter of the stented segment was significantly smaller in diabetic group than in control group, as well as percentage of intimal hyperplasia at IVUS analysis, resulting in significantly increased late lumen loss in diabetic group (Table 3). No significant differences were observed of angiographic and IVUS measurements between the left and right coronary system in diabetic and control animals, and no stent- related thrombosis formation was found in both groups.

**Table 3:** Angiographic and IVUS Follow-up Results

	Diabetic group (n = 15)	Control group (n = 15)
In-stent MLD(mm)	2.45 ± 0.32*	2.82 ± 0.21
In-segment MLD(mm)	2.43 ± 0.29*	2.79 ± 0.27
In-stent DS(%)	40.4 ± 24.0*	20.2 ± 17.7
In-segment DS(%)	43.1 ± 23.9*	21.3 ± 15.7
In-stent LL(mm)	0.33 ± 0.19*	0.10 ± 0.09
In-segment LL(mm)	0.31 ± 0.18*	0.10 ± 0.15
In-stent restenosis > 50% (number of stent)	6	2
Neointimal volume(mm^3^)	21.9 ± 18.7**	3.87 ± 2.89
%IH	26.7 ± 19.2**	7.3 ± 6.1

IVUS = intravascular ultrasound, MLD = minimal lumen diameter, DS = diameter stenosis, LL = late loss, %IH = percentage of intimal hyperplasia. *p < 0.05, **p < 0.001 vs. control group.

### Histological findings

**Figure 1:**
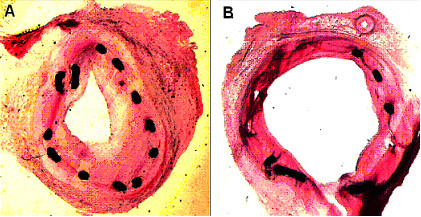
Micrographs of stented left anterior descending arteries in diabetic (A) and control (B) porcine with HE staining (× 10 objective).

**Figure 2:**
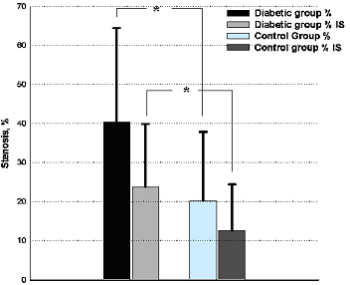
Uncorrected (%) and corrected (%IS) percentage of stenosis between the two groups. * p < 0.05, IS = injury score.

The degree of intimal injury caused by stent deployment was similar between the two groups. Significantly aggravated intima hyperplasia was observed in diabetic group by morphometric analysis with parameters of percentage of stenosis, intima area, medial area and maximal intimal thickness (Table 4). The photomicrograph of the intimal proliferative response to stent implantation in the diabetic and control groups was shown in Figure 1A and 1B. Uncorrected and corrected percentage of stenosis between the two groups was displayed in Figure 2.

**Table 4:** Results of histological analysis

	Diabetic group (n = 15)	Control group (n = 15)
IS	1.68 ± 0.19	1.73 ± 0.13
Lumen area(mm^2^)	4.47 ± 2.56*	5.85 ± 2.07
Area within IEL(mm^2^)	6.19 ± 0.93	6.23 ± 1.01
Neointimal area(mm^2^)	1.59 ± 0.76 *	0.41 ± 0.18
MIT(mm)	1.24 ± 0.76*	0.59 ± 0.28

IS = injury score; IEL = internal elastic limina; EEL = external elastic lamina; MIT = maximal intima thickness. *p < 0.05**p < 0.001 vs control grpup.

### Inflammatory cytokines in aortic wall

**Figure 3:**
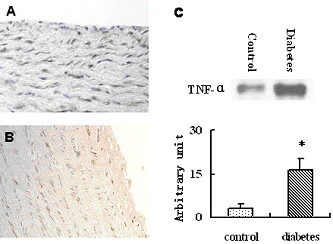
Immunohistochemistry and Western blotting of TNF-α (×40 objective). TNF-α expression in aortic intima tissue of diabetic (A) and control (B) porcine. C, TNF-α levels quantified in conditioned medium of aortic intima. *p < 0.001.

**Figure 4:**
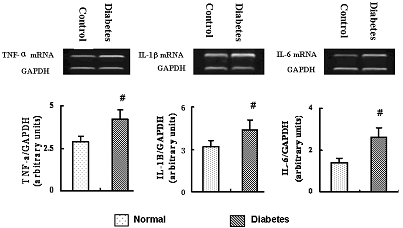
TNF-α, IL-1 and IL-6 mRNA in aortic intima of diabetic porcine and controls. # P < 0.05.

Compared to controls, dramatic elevation in TNF-α levels was revealed in aortic intima of diabetic group (p < 0.001, Fig. 3). The result was supported by immunohistochemical assessment, which displayed more TNF-α expression in diabetic porcine aorta (Fig. 3). RT-PCR examination demonstrated significantly higher levels of TNF-α, IL-1β and IL-6 mRNA in aortic intima of diabetic porcine, as comparing to the controls (Fig. 4), which was also parallel to the results of Western blotting.

## Discussion

The current study indicates that diabetic state attenuated the anti-proliferation effects of SES as comparing to the non-diabetic counterpart. Angiographic and IVUS follow-up at 6 months, as well as histological examinations after animal sacrifice demonstrated a significant increase in late lumen loss and reduction of lumen area caused by exacerbated neointimal hyperplasia after coronary stenting in the diabetic porcine. An exaggerated inflammatory response within the aortic wall of the diabetic model was proved by Western blotting and RT-PCR, similar to the results previously reported [18]. Enhanced expression of proinflammatory mediators in diabetic vasculature may be linked to multiple mechanisms germane to progression of in-stent restenosis, such as smooth muscle cell migration and arterial remodeling.

### Coronary restenosis in diabetic porcine model

Previous preclinical studies of drug-eluting stents mainly assessed their safety more than efficacy in the non-diabetic settings. In a non-diabetic porcine model, Suzuki et al reported that SES reduced in-stent neointimal proliferation at 28 days, compared to the bare metal stent [24]. As drug-eluting stents have recently been widely applied to majority of diabetic patients during percutaneous coronary intervention in routine clinical practice, determination of the inhibitory effect of SES on neointimal growth in a diabetic animal model may be more relevant to the understanding of mechanism for an increased restenosis rate seen in the clinical diabetic cohort, although neointimal proliferation was supposed to be same as in bare-metal stent studies[19]. While increased neointimal hyperplasia was shown in the diabetic rat aorta after implantation of drug-eluting stent [25], the changes of coronary neointimal proliferation in large animal models remain unclear.

In this study, we successfully established STZ-induced diabetic porcine model. Minipigs were chosen because their coronary anatomy and vascular structure were thought to be more ideal for stent implantation than other animal models in the assessment of this device. In order to avoid the influence of coronary distribution, two drug-eluting stents have been deployed into two major epicardial coronary arteries for each animal, and left anterior descending artery and right coronary artery were preferentially stented, as restenosis was more common in these arteries than in left circumflex artery [26,27]. The final stent-to-vessel diameter ratio ranged 1.1~ 1.2:1 in the current study, which could generally create sufficient coronary injury to induce neointimal hyperplasia [28].

In general, neointimal response is exaggerated and the time course of healing is more prolonged in humans than in animals [29]. In a porcine model, peak neointimal growth after implantation of bare metal stents is observed at 28 days, compared with 6–12 months in humans [19,30]. The precise time course of peak neointimal growth in human coronary arteries after drug-eluting stent implantation remains not fully defined. Previous animal studies have shown favorable results of 28 days after drug-eluting stent deployment, with a lack of sustained efficacy at 3 and 9 months [24]. Previous studies have found that the degree of neointimal hyperplasia peaked at 6 months after intervention in the coronary arteries followed by regression in some extent [30,31]. The findings at 6 months when the injury healing process is completed in animals, may stand for the results of two to three years in human [30]. Therefore, we assessed the 6-month angiographic and IVUS results in the diabetic porcine after receiving SES implantation. Our study indicates that in the diabetic animals, the degree of neointimal proliferation was significantly exaggerated than that in the control group, with respect to increased in-stent late lumen loss at angiography (0.33 ± 0.19 mm vs. 0.10 ± 0.09 mm, p < 0.05) and percentage of volume obstruction by IVUS imaging (26.7% ± 19.2% vs. 7.3% ± 6.1%, p < 0.001) during 6-month follow-up. The latter has been regarded as an accurate index for evaluating the efficacy of neointimal inhibition in drug-eluting stent era [32]. Furthermore, histological findings after animal sacrifice were also consistent with the angiographic and IVUS results. After correction of the injury score, the percentage of lumen area stenosis was significantly increased in diabetic group (23.7% ± 17.4% vs. 11.9% ± 13.2% in control group, p < 0.05).

### Mechanism of exaggerated vascular response in diabetic porcine

The mechanisms of neointimal hyperplasia after coronary stenting with SES are complex, involving damage of vessel medium, endothelial laceration, platelet aggregation and activation, and release of tissue growth factors [30]. In diabetes, metabolic abnormalities and hemodynamic changes may occur, including hyperglycemia, insulin-resistance, hyperlipidemia, which may lead to hypertension, endothelial dysfunction, and atherosclerotic plaque formation [33,34]. In addition, evidence is mounting that an exaggerated inflammatory response within the blood vessel wall contributes to acceleration of vascular lesion area and complexity. The magnitude of vascular inflammatory response to injury after coronary stenting may play a central role for the degree of neointimal proliferation and correlated with adverse late clinical outcomes (death, myocardial infarction, recurrent ischemia, and restenosis) [35]. Clinical studies in support of the anti-inflammatory effects of abciximab and statins by modulating atherosclerotic plaque stability have been reported [5].

Our results strongly suggest that exaggerated neointimal hyperplasia after SES implantation in the diabetic porcine may be related to an enhanced inflammatory response to vascular injury. In this study and our previous report with less amount of diabetic animal models [18], a great elevation of mRNA levels of cytokine TNF-α, IL-1β and IL-6 and a dramatic increase of TNF-α secretion in the aortic wall of diabetic porcine, as comparing to the non-diabetic animals, may be caused by activation of inflammation-related pathways and regulators in vessel wall driven by hyperglycemia, oxidative stress and formation of advanced glycated end products, further supporting the concept that diabetes mellitus is an independent and potent risk factor for atherosclerosis [6,36]. Pathophysiological features detected in aorta properly represent general phenomenon in large as well as medium size arteries [34]. Nevertheless, it would be worthwhile to evaluate the degree of inflammation in the non-stented branch of coronary vasculatures as well. Our results indicate that enhanced inflammatory response in diabetic cohort may also have contributed to the development of neointimal hyperplasia even after drug-eluting stent implantation, although their precise relationship still needs further investigation.

In this study, we examined the inflammatory mediators in the aortic intima to exclude the interference from polymer-induced inflammation in the coronary arteries. The non-reabsorbable polymer loaded on the drug-eluting stents would cause local chronic inflammation and lead to increased neointimal hyperplasia and late stent thrombosis [35]. As vascular inflammation represents the "final common pathway" for many disease processes, it may, thus, represent the "ultimate therapeutic target" for pharmacologic inhibition. In this context, emphasis should be made with regard to the use of anti-inflammatory agents in the prevention of neointimal hyperplasia after implantation of SES in clinical practice.

## Limitations

Although animal model of restenosis has been widely used to evaluate the safety and efficacy of coronary stents, the accuracy of reflecting the human coronary artery response to such stents is still unclear. STZ was used to induce diabetes in the current study, which results in a type 1 diabetic model with injury of pancreas; therefore, the term from induction of diabetes to stent implantation is limited in this study. It has recently been demonstrated that patients with insulin-dependent diabetes had higher in-stent late lumen loss compared to non-insulin dependent diabetic patients. Hyperinsulinemia was reported to enhance neointimal hyperplasia in the rat carotid injury model through activation of ras/MARK pathway [37], but in a human study, insulin levels showed no significant association with restenosis [38]. Likewise, only male animals were used, therefore, gender-related differences in the rate of restenosis in diabetic state or in the use of SES remain unclear. Furthermore, inflammatory cytokines were measured using conditioned media of the aortic intima, it would be interesting to evaluate serum levels of these inflammatory markers, which could monitor the time course of the inflammatory activity in this experimental model. Finally, results from animal studies may not translate to patients, due to improper or biased variable selection, or confounding effects of vascular injury [19].

## Conclusion

Neointimal hyperplasia persisted at 6 months after SES implantation in the diabetic porcine model, which may be, at least partly, related to an exacerbated inflammatory response within the diabetic blood vessel wall. Further studies are needed to assess the molecular mechanism of enhanced neointimal proliferation and effects of pharmacological intervention.
